# Prognostic analysis of lung adenocarcinoma based on cancer-associated fibroblasts genes using scRNA-sequencing

**DOI:** 10.18632/aging.204838

**Published:** 2023-07-11

**Authors:** Han Zhang, Yuhang Wang, Kai Wang, Yun Ding, Xin Li, Shuai Zhao, Xiaoteng Jia, Daqiang Sun

**Affiliations:** 1 Clinical School of Thoracic, Tianjin Medical University, Tianjin, China; 2 Department of Thoracic Surgery, Tianjin Chest Hospital of Tianjin University, Tianjin, China

**Keywords:** scRNA-seq, CAFs markers, prognosis models, immunotherapy, drug sensitivity

## Abstract

Cancer-associated fibroblasts (CAFs) are an important component of the tumor microenvironment (TME). CAFs can promote tumor occurrence and metastasis by promoting cancer cell proliferation, angiogenesis, extracellular matrix (ECM) remodeling, and drug resistance. Nevertheless, how CAFs are related to Lung adenocarcinoma (LUAD) has not yet been revealed, especially since the CAFs-related prediction model has yet to be established. We combined Single-cell RNA-sequencing (scRNA-seq) and Bulk-RNA data to develop a predictive model of 8 CAFs-associated genes. Our model predicted LUAD prognosis and immunotherapy efficacy. TME, mutation landscape and drug sensitivity differences were also systematically analyzed between the LUAD patients of high- and low-risk. Moreover, the model prognostic performance was validated in four independent validation cohorts in the Gene expression omnibus (GEO) and the IMvigor210 immunotherapy cohort.

## INTRODUCTION

Lung cancer has the highest prevalence and mortality among all malignancies [1], with 2.1 million diagnosed cases yearly, resulting in 1.8 million deaths [2]. Lung cancer comprises non-small (NSCLC; > 85%) and small cell lung cancer (SCLC; < 15%) [3]. LUAD is the primary NSCLC type [4]. Surgical resection offers the most efficient treatment option for LUAD patients at the early stage [5]. Furthermore, several therapies, including chemotherapy, targeted therapy, radiotherapy, and immunotherapy, have significantly enhanced LUAD patient survival rates [6]. LUAD treatment has significantly improved, while the prognosis remains dismal, especially for advanced cases [7]. Immune checkpoint inhibitors (ICIs) for PD-1, PD-L1, and CTLA4 have changed the treatment mode in advanced NSCLC. However, immunotherapy efficiency is limited [8], and in some cases, it leads to more rapid tumor growth than the patients who did not receive immunotherapy [9]. In summary, there are still many challenges in treating LUAD, and we must further investigate prognostic and efficacy predictors.

TME is the dynamic and highly heterogeneous environment in which cancerous cells interact with their surroundings, including immune cells, stromal cells, and blood vessels [10]. It has been demonstrated that CAFs are essential for TME [11–13]. CAFs can promote tumor occurrence and metastasis by promoting cancer cell proliferation, angiogenesis, ECM remodeling, and drug resistance [11, 14, 15]. CAFs in lung cancer upregulate *MiR-21* to induce calumenin protein secretion, thereby increasing tumor aggressiveness [16]. Detailed studies of the CAFs-immune microenvironment interactions, especially the CAFs-immune cell sophisticated mechanisms, may yield innovative approaches to target CAF-directed immunotherapy in the future [17]. The role of CAFs in LUAD has yet to be investigated.

TME cannot be characterized at high resolution using current bulk omics analyses. scRNA-seq facilitates massively parallel characterization of multiple cells at the transcriptome level, compensating for the limitations of traditional bulk-omic to explore TME characteristics more deeply and comprehensively [18–20]. Previous studies combining scRNA-seq and bulk RNA-seq successfully established prediction models to predict LUAD prognosis and immunotherapy efficacy based on B cells and natural killer (NK) cells [21, 22]. This study aims to analyze scRNA-seq data from 10 untreated primary LUAD cases systematically and identify 120 genes associated with CAFs. Finally, an 8-gene prognostic model was designated utilizing Lasso and stepwise multivariate COX regression on the above gene sets in The Cancer Genome Atlas (TCGA) database. TME, mutation landscape and drug sensitivity differences were systematically analyzed between the LUAD patients of high- and low-risk. Moreover, the model prognostic performance was validated in four independent validation cohorts in the GEO and the IMvigor210 immunotherapy cohort.

## MATERIALS AND METHODS

The scRNA-seq files of 10 LUAD patients were downloaded from https://codeocean.com/capsule/8321305/tree/v1. The RNA sequencing profiles and clinical and mutation information of 517 LUAD patients were obtained from The Cancer Genome Atlas (TCGA; https://portal.gdc.cancer.gov/). The University of California, Santa Cruz, Xena browser (UCSC Xena; https://xenabrowser.net/) was also accessed to download LUAD patient survival data as a supplement. Additionally, GEO (http://www.ncbi.nlm.nih.gov/geo/) was assessed, and four microarray data, GSE31210 (n = 246), GSE37745 (n = 196), GSE50081 (n = 181), and GSE72094 (n = 442), were downloaded. The LUAD patient Tumor Immune Dysfunction and Exclusion (TIDE) scores were downloaded by accessing http://tide.dfci.harvard.edu/. Immunophenoscore (IPS) data of LUAD patients were downloaded from the Cancer Immunome Atlas (TCIA) database (http://tcia.at/), which is positively related to cancer immunogenicity with the predictive ability of the cancer patient immunotherapy response [23]. The immunotherapeutic cohort (IMvigor210) was acquired according to the guideline at http://research-pub.gene.com/IMvigor210CoreBiologies/ [24].

### Cancer-associated fibroblasts (CAFs) marker gene identification

‘Seurat (version 4.2.0)’ R package was utilized to read and quality control scRNA-seq files. The primary steps were as follows: The function Read-10x was used to read the data, and the function ‘CreatSeuratObject’ was used to create the object. Then, the data were merged, and low-quality cells were removed with nFeature > 10000 or < 500, nCount > 100000 or < 1000, mitochondrial gene > 30%, and erythrocyte gene > 5%. Afterward, variance-stabilized UMI counts were normalized using the ‘SCTransform’ function [25]. SNN graphs and UMAP embeddings were constructed using the top 30 principal components. A canonical cell type marker score was used to identify the cell types in cell clusters. Markers were computed for each cell cluster using the ‘FindAllMarkers’ function with the following parameters: only.pos = T, min.pct = 0.25, logfc.threshold = 1. Thus, 120 marker genes related to CAFs were identified (Supplementary Table 1). The association was analyzed between individual cell subsets and 50 Hallmarkers by performing the R-package ‘singleseqgset (version 1.1.0)’. The related gene expressions were also visualized using the ‘FeaturePlot’ function in different cell subsets.

### Prognostic signature based on CAFs marker genes construction and validation

The ‘survival (version 3.3-1)’ and ‘survivalminer (version 0.4.9)’ R packages were used to perform a univariate COX regression for CAFS-related genes, with 32 genes exhibiting significant effects on overall survival (OS). Thirteen genes were selected for the Cox regression model using the ‘glmnet (version 4.1-4)’ R package and the minimum absolute contraction and LASSO method. Further gene screening was conducted using stepwise multiple COX regression for OS genes. Finally, a risk model was created based on eight gene mRNA expressions and their associated risk coefficients. Based on the median model score (4.842808), the TCGA patients were separated into high- and low-risk groups for each GEO-independent validation set. Kaplan-Meier was applied for the high-risk group survival analysis, and using the ‘timeROC (version 0.4)’ R package, the area under the curve (AUC) was calculated. The R package ‘regplot (version 1.1)’ was utilized for building the nomogram. Simultaneously, the model diagnostic capability was verified using four independent GEO datasets.

### Enrichment analysis of CAFs

Besides converting the Gene ID into EntrezID, the 120 CAFS-related gene expression levels of LUAD patients were extracted from the TCGA database. Gene Ontology (GO) [26] and Kyoto Encyclopedia of Genes and Genomes (KEGG) [27] were employed for the risk groups using the ‘clusterProfiler (version 4.4.4)’ R package.

### Somatic mutations analysis

The ‘maftools (version 2.12.05)’ R package was utilized to calculate and display the top 20 gene mutation landscape using TCGA database-tumor mutation data. The difference in tumor mutation burden (TMB) between the risk groups was illustrated as a violin plot. The Kaplan-Meier method revealed that high- and low-TMB and risk scores impact survival outcomes.

### Estimate of tumor immune microenvironment

The TCGA database immune-infiltrating files for all tumors were acquired using the TIMER2.0 database (http://timer.cistrome.org). Then, the Wilcoxon rank-sum was applied to calculate the difference in immune cell infiltration between six algorithms, including TIMER [28], CIBERSORT [29], CIBERSORT-ABS [30], QUANTISEQ [31], MCPCOUNTER [32], XCELL [33], and EPIC [34]. The above process was implemented using the ‘limma v 3.52.4’ and ‘pheatmap (v 1.0.12)’ R packages. Next, immune checkpoint expression and major histocompatibility complex (MHC) gene were compared between the two risk patients using the same method. Supplementary Table 2 depicts these genes. Other cancer-related gene sets, such as chemokines, growth factors and regulators, proteases and regulators, soluble or shed receptors or ligands, and interleukins, were also explored for differential expression between the two groups. Finally, the R package ‘estimate’ was conducted to further evaluate the LUAD patient immune microenvironment.

### Immunotherapy efficacy prediction

The patient IPS was calculated to predict the immunotherapy response under various conditions. Next, the risk score ability was validated to predict the immunotherapy response in the IMvigor210 immunotherapy cohort. Finally, the risk group TIDE score was compared, revealing a positive correlation with the possibility of immune escape.

### Prediction of chemotherapy response

The LUAD chemotherapy response data were downloaded from the Genomics of Drug Sensitivity in Cancer database (GDSC, https://www.cancerrxgene.org) [35]. The R package ‘oncoPredict (version 0.2)’ was utilized to analyze the two-group sensitivity to various chemotherapy drugs. Additionally, R packages ‘ggplot2 (version 3.4.0)’ and ‘ggpubr (version 0.5.0)’ were utilized to visualize the results.

### Data availability statement

All data generated or analyzed during this study are included within this article.

## RESULTS

### Identification of CAFs marker genes

**Figure 1:**
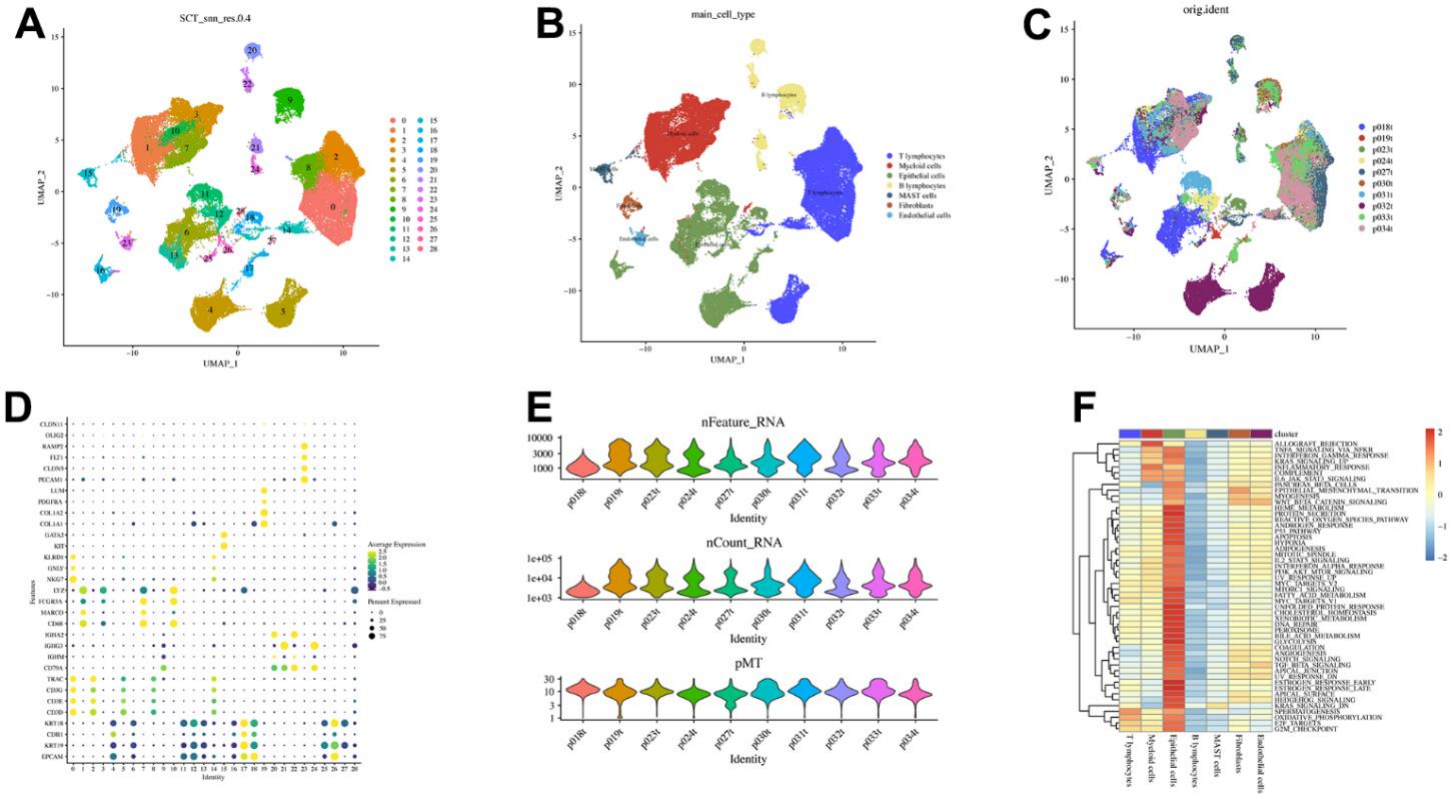
**Visualization of scRNA-seq data from 10 LUAD patients.** (**A**) The U-MAP algorithm identified 29 cell subsets. (**B**) Seven cell types were identified based on marker genes. (**C**) UMAP plot of 62,115 cells, colored by patients. (**D**) Marker genes for different cell subsets. (**E**) Quality control results. (**F**) Association between cell subsets and Hallmarkers.

We obtained single-cell sequencing data from 10 surgically resected primary LAUDs (73,566 cells) without specific treatment [36]. After strict quality control (removing nFeature > 10,000 or 500, nCount > 100,000 or 1,000, mitochondrial gene > 30%, and erythrocyte gene > 5%), 62115 high-quality cells were obtained. Figure 1E depicts the quality control results for each patient as a violin plot. The SCTransform algorithm identified 3,000 hypervariable genes and data homogenization and normalization. For the 3,000 genes mentioned above, 30 PCs were used to reduce dimensionality, yielding 29 cell clusters (Figure 1A). Subsequently, annotation with canonical cell markers (Figure 1D) yielded seven cell subsets, with the 19th cluster annotated as CAFs. However, the cell distribution existed between different patients, but there was no significant batch effect (Figure 1C). Analysis of seven cell subsets and 50 Hallmarkers revealed a significant positive correlation between CAFs and epithelial-mesenchymal transition (EMT), myogenesis, and Wnt/β-catenin signaling (Figure 1F). Ultimately, the ‘FindAllMarkers’ function yielded 120 CAFs-related genes.

### Prognostic signature based on CAFs marker genes construction and validation

Univariate COX regression analysis of 120 CAFs-related genes in the TCGA database revealed that 32 genes were significantly related to OS, with seven genes representing protective factors and 25 genes representing risk factors (Supplementary Figure 1A). These 32 genes were further analyzed using LassoCOX regression, and 13 were obtained according to the optimal λ value (logλ = -4). Then, the abovementioned 13 genes were used for stepwise multivariable COX regression analysis. Finally, eight genes, *TIMP1*, *TPM2*, *NR2F2*, *MFAP4*, *SOD3*, *CAV1*, *SERPINH1*, and *FMO2*, were used to build a prognosis model. The ‘Featureplot’ function was applied to display these eight gene expressions in different cell subsets. Online TIMER (http://timer.comp-genomics.org) was accessed to investigate these eight abnormal gene expressions in pan-cancer (Supplementary Figure 1D, 1E). The final model combined with the coefficient of each gene is: Risk = (0.2403052*TIMP1 expression) + (0.1475574*TPM2 expression) + (0.2686883*NR2F2 expression) + (-0.1635410*MFAP4 expression) + (-0.1219534*SOD3 expression) + (0.2009316*CAV1 expression) + (0.1449876*SERPINH1 expression) + (-0.1816038*FMO2 expression).

**Figure 2:**
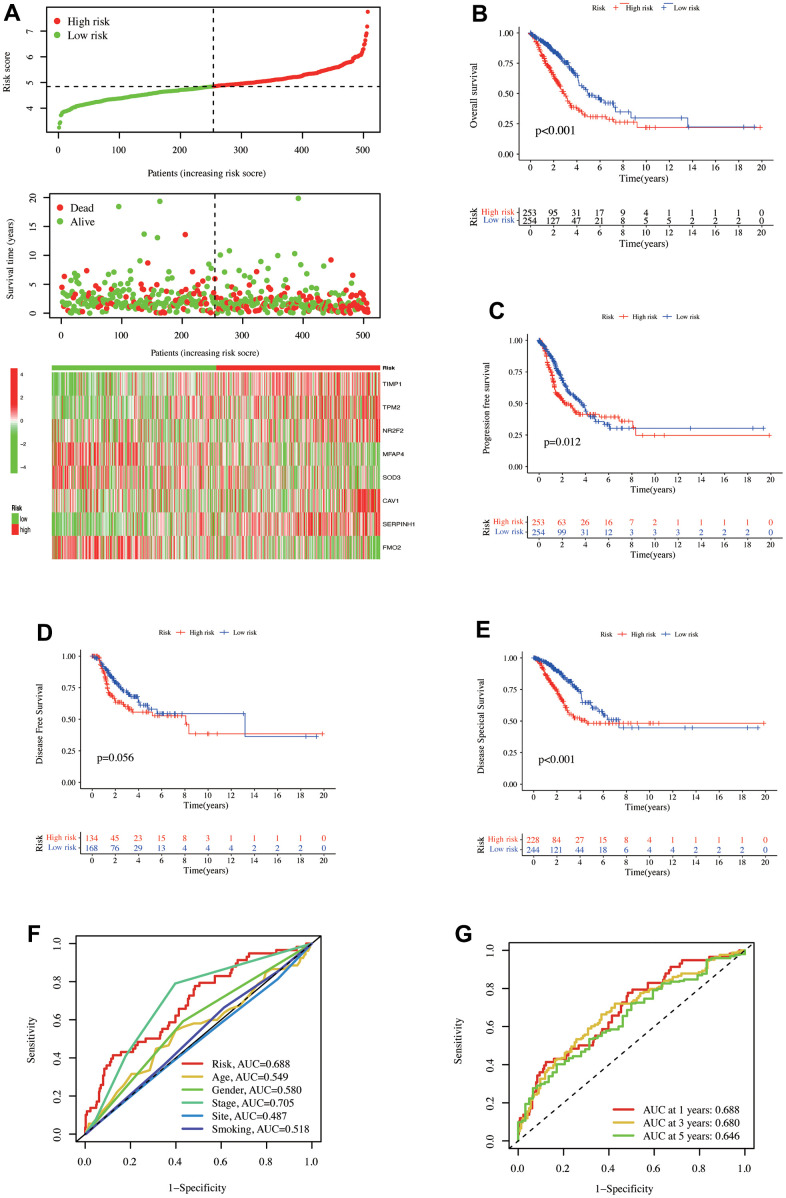
**The prediction model of CAFs-related genes was established in the TCGA database.** (**A**) Distribution of risk scores and OS status. 8 gene expressions were involved in the model construction in the high- and low-risk groups. Kaplan-Meier curve illustrated the differences between high- and low-risk groups for (**B**) OS, (**C**) PFS, (**D**) DFS, and (**E**) DSS. (**F**) ROC curves for risk scores and clinical characteristics. (**G**) ROC curves for one-, three-, and five-year OS of risk scores in the TCGA database.

An increased risk score indicates an increase in the number of patients who have died, indicating a high risk and poor OS positive association. The high-risk group had significant *TIMP1*, *TPM2*, *NR2F2*, *CAV1*, and *SERP1NH1* expressions, whereas the low-risk group had significant *MFAP4*, *SOD3*, and *FMO2* expressions (Figure 2A). The Kaplan-Meier (K-M) survival curve demonstrated that high-risk patients had significantly lower OS (p < 0.001, Figure 2B), PFS (p = 0.012), and DSS (p < 0.001, Figure 2C, 2E). Despite this, the two groups did not differ significantly in DFS (p = 0.056). The ‘timeROC’ R package was employed to evaluate the model diagnostic efficacy and related clinical characteristics for survival outcomes, revealing that pathological stage (AUC = 0.705) and risk (AUC = 0.688, Figure 2F) were the two indicators with good diagnostic efficiency. After 10-fold cross-validation, the model’s performance was evaluated, and its mean AUC values were 0.688, 0.680, and 0.646 over one, three, and five years, respectively.

**Figure 3:**
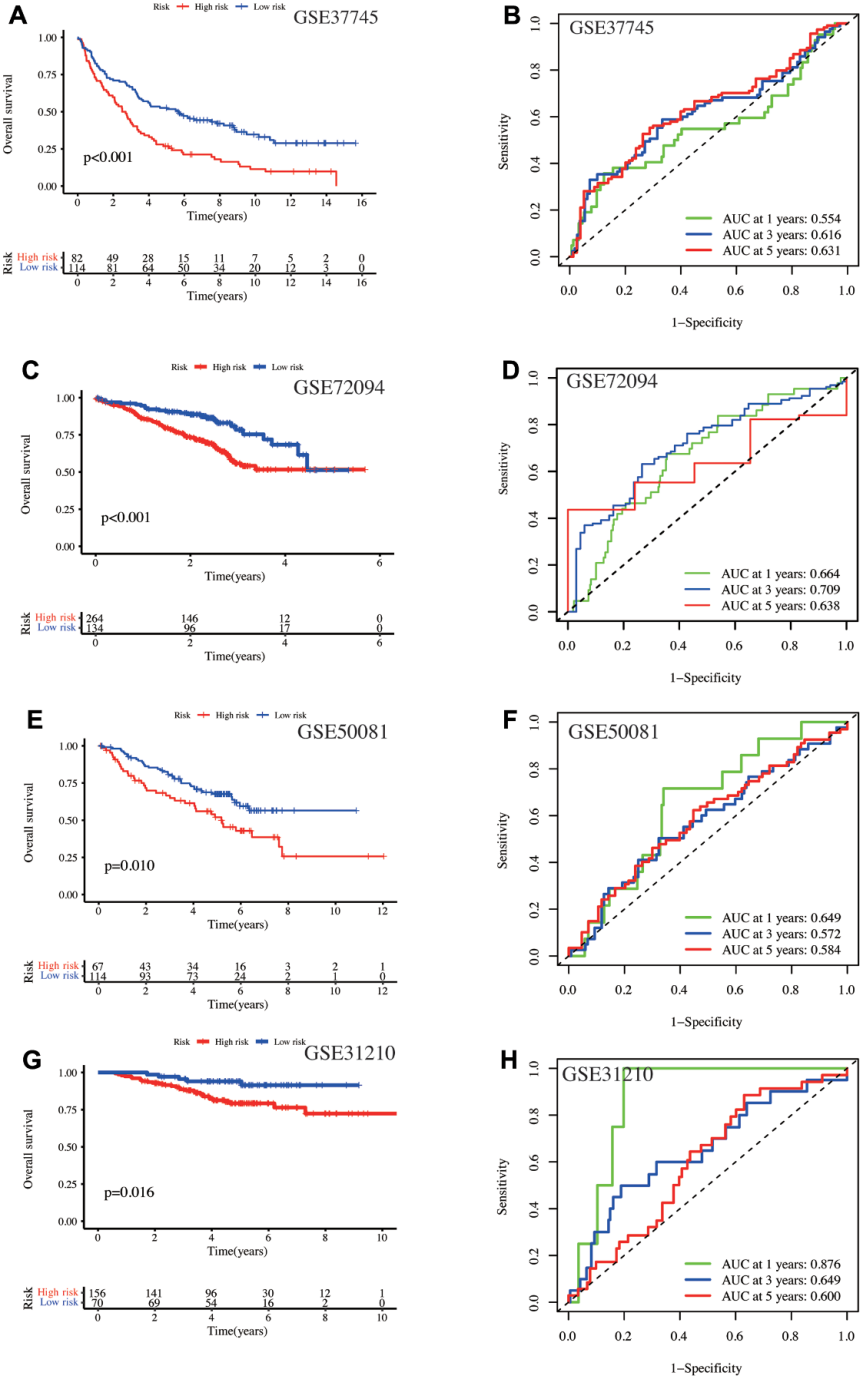
**Validation of the model using four independent GEO database cohorts.** In the GSE37745 cohort, (**A**) Kaplan-Meier curves depict the prognosis for the high- and low-risk groups, and (**B**) the AUC values at one-, three-, and five-year. In the GSE72094 cohort, (**C**) Kaplan-Meier curves show the prognosis of the high- and low-risk groups, and (**D**) the AUC values at one-, three-, and five-year. In the GSE50081 cohort, (**E**) K-M curves display the prognosis for the high- and low-risk groups, and (**F**) the AUC values at one-, three-, and five-year. In the GSE31210 cohort, (**G**) Kaplan-Meier curves depict the prognosis of the high- and low-risk groups, and (**H**) the AUC values at one-, three-, and five-year.

Next, four independent GEO datasets verified the model’s validity and stability. In GSE37745, the high-risk patient had a worse prognosis (p < 0.001, Figure 3A), with AUC values of 0.554, 0.616, and 0.631 at one-, three-, and five-year, respectively (Figure 3B). In GSE72094, the low-risk patient OS was significantly prolonged (p < 0.001, Figure 3C), with AUC values of 0.664, 0.709, and 0.638 at one-, three-, and five-year, respectively (Figure 3D). In GSE50081, high-risk patients had poor OS (p = 0.01, Figure 3E), with AUC values of 0.649, 0.572, and 0.584 at one-, three-, and five-year, respectively (Figure 3F). In GSE31210, the high-risk patient had poor OS (p = 0.016, Figure 3G) and AUC values of 0.876, 0.649, and 0.600 with the one-, three-, and five-year, respectively (Figure 3H). In conclusion, high-risk patients had poor long-term survival outcomes in validation and training sets. Furthermore, our model has a good prediction efficiency for the patient survival outcome.

### The model diagnostic and prognostic ability combined with clinical features

**Figure 4:**
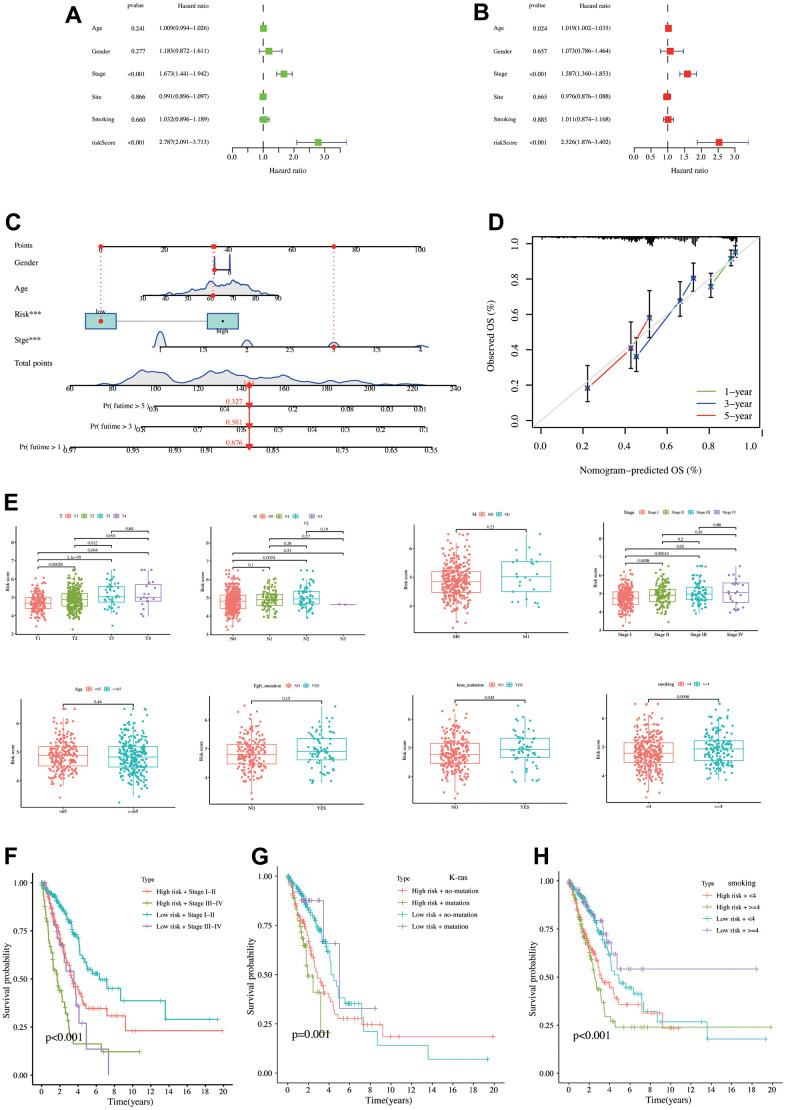
**Establishment and validation of nomograms, risk score differences between different clinical characteristics.** (**A**) Univariate COX regression analysis. (**B**) Multivariate COX regression analysis. (**C**) Nomogram constructed using risk score and clinical characteristics. (**D**) Calibration curve of the nomogram. (**E**) Risk score differences between different clinical characteristics. The K-M analysis curves for the patients stratified by (**F**) risk score and stage, (**G**) risk score and K-Ras mutation status, (**H**) risk score, and smoking level.

Univariate COX regression was applied in the TCGA database by five clinical features, including age, gender, stage, tumor site, and smoking status. Besides the model risk score, OS risk factors revealed stage (p < 0.001, HR = 1.763, 95% CI = 1.441–1.942) with risk score (p < 0.001, HR = 2.787, 95% CI = 2.091–3.713, Figure 4A). Meanwhile, Multivariate COX regression findings revealed that the OS risk factors were age (p = 0.024, HR = 1.019, 95% CI = 1.002–1.035), stage (p < 0.001, HR = 1.587, 95% CI = 1.360–1.853), and risk score (p < 0.001, HR = 2.526, 95% CI = 1.876–3.402, Figure 4B). The model risk score was a survival outcome risk factor in both COX analyses. Next, the constructed nomogram revealed that a patient’s gender, age, risk score, and pathology stage are as follows: male, 60 years old, low risk, and stage III. Then, the patient’s total score was 144, and the one-, three-, and five-year survival rates were 0.876, 0.581, and 0.327, respectively (Figure 4C). The calibration curve exhibited a high correlation between predicted and actual values, revealing good predictive nomogram performance (Figure 4D). Figure 4E demonstrates that patients with a later pathological stage had a higher risk score, partly explaining the poor prognosis of the high-risk patient. Risk scores did not differ significantly between patients aged 65 years and those aged more than or less than 65 years (p = 0.44) and between EGFR mutation and non-mutation groups (p = 0.15). Patients with a K-RAS mutation exhibited a higher risk score (p = 0.043), as did patients with a higher smoking score (p = 0.0098). Next, patients were stratified using the statistically significant clinical characteristics and risk scores described above. High-risk + stage III−IV exhibited a poor prognosis, while low-risk + stage I−II exhibited the best prognosis (Figure 4F). Meanwhile, the K-RAS mutation + high-risk group had poor survival prognosis than the low-risk and K-RAS non-mutation groups (Figure 4G). Finally, the high-risk + high-smoking score patients also have a poor survival prognosis (Figure 4H).

### Enrichment analysis

**Figure 5:**
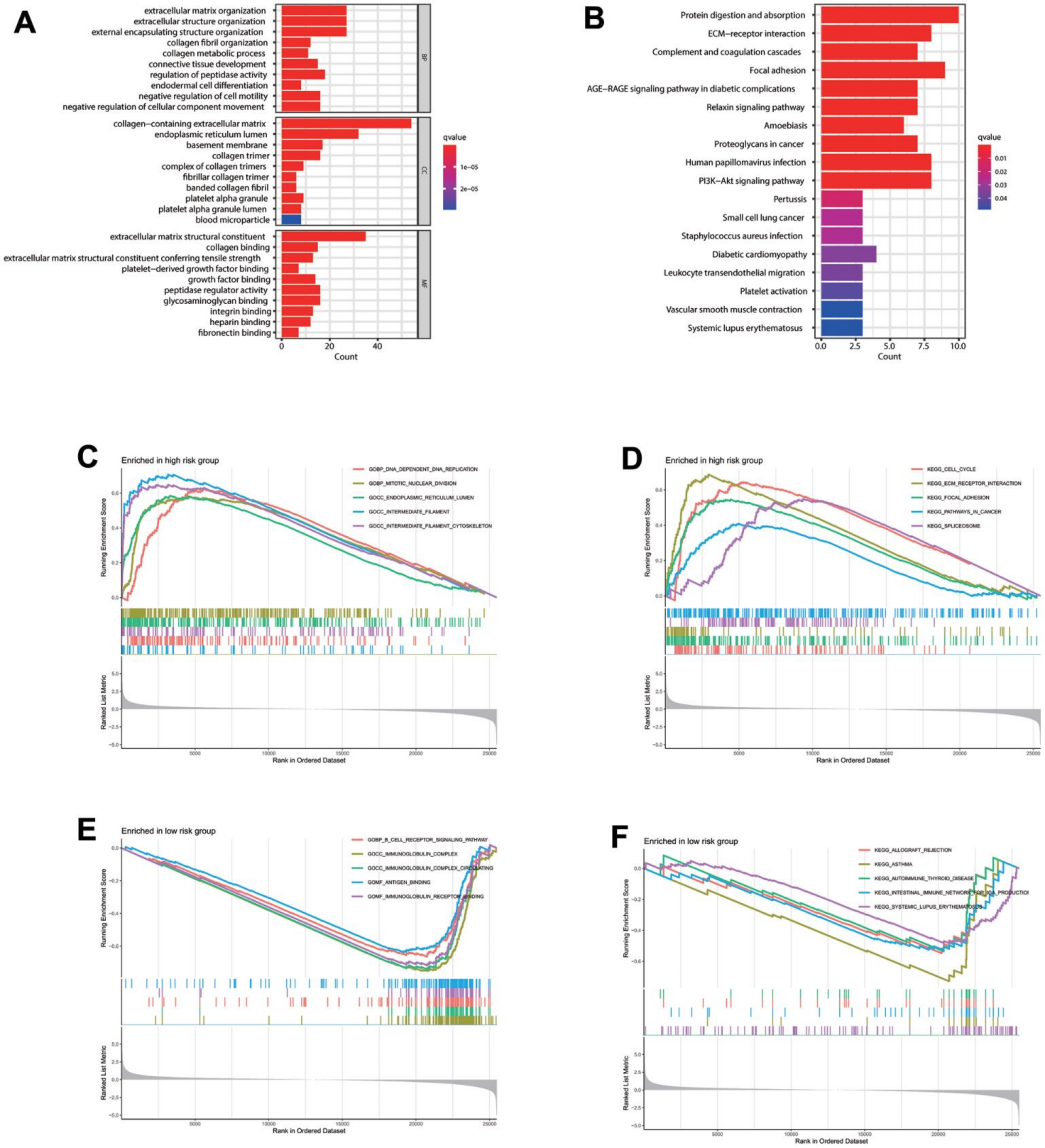
**Enrichment analysis.** (**A**) GO and (**B**) KEGG enrichment analysis of 120 CAFs-related genes. Gene Set Enrichment Analysis (GSEA) analysis for patients in the (**C**, **D**) high- and (**E**, **F**) low-risk groups.

GO analysis indicated the significance of the 120 CAFs-related genes enrichment in ECM organization and structural constituent besides the collagen-containing ECM and other pathways (Figure 5A). Moreover, KEGG analysis indicated that these genes significantly correlate with protein digestion and absorption, ECM-receptor interaction, complement and coagulation cascades, and other pathways (Figure 5B), indicating the CAF importance in ECM remodeling, consistent with former studies [13–15]. KEGG and GO analyses were also conducted to explore functional differences between the risk groups. According to Gene Set Enrichment Analysis (GSEA) analysis, the significant high-risk group enrichment in DNA replication, mitotic nuclear division, and other pathways (Figure 5C) indicates its significant correlation with cell replication pathways. Meanwhile, the significant low-risk group enrichment in the B cell receptor (BCR) and immunoglobulin complex pathways (Figure 5E) indicates its significant enrichment in immune-related pathways. GSEA analysis also indicated the significant enrichment of cell cycle, ECM receptor interaction, and focal adhesion pathway in the high-risk group (Figure 5D). Meanwhile, the low-risk group enrichment appeared in allograft rejection, asthma, and autoimmune thyroid disorder pathway (Figure 5F).

### Somatic mutation analysis

**Figure 6:**
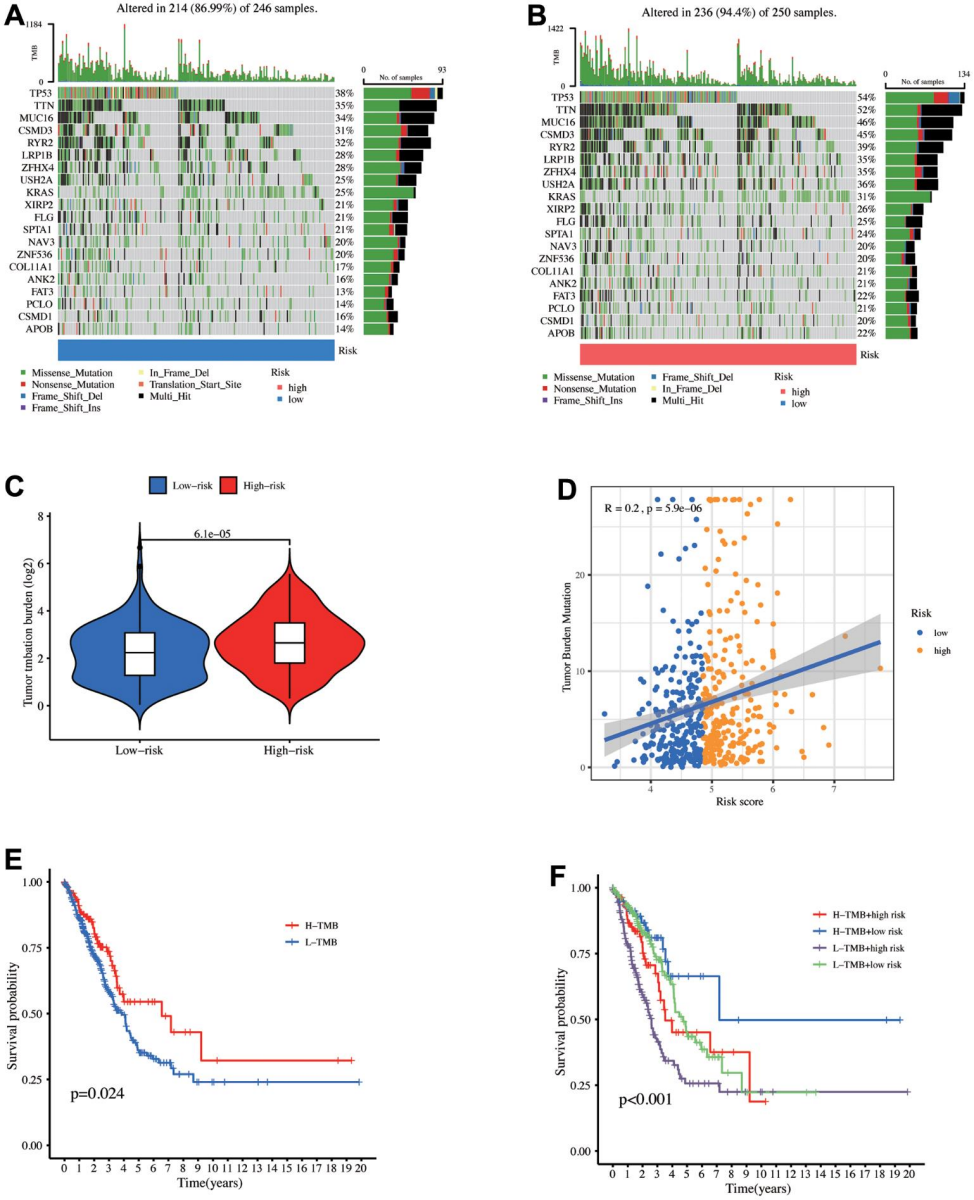
**Mutation analysis between the high- and low-risk groups.** The mutational landscapes of the (**A**) high- and (**B**) low-risk groups. (**C**) TMB level comparison between high- and low-risk groups. (**D**) Correlation between risk score and TMB. (**E**) K-M curves for high and low TMB levels. (**F**) K-M curves for the patients stratified by risk score and TMB.

TMB is a quantitative biomarker that reflects the total tumor cell mutation count, the total count of somatic gene coding errors, base substitution, gene insertion, or deletion errors detected per million bases [37]. Higher TMB levels correlated with longer OS after immunotherapy across multiple cancers. Figure 6A, 6B depict a waterfall map of the top 20 genes mutation landscape with the highest mutation frequency. The high-risk patients exhibited increased TMB levels (p = 6.1e-05), suggesting their further benefit from the immunotherapy (Figure 6C). Meanwhile, Figure 6D depicts that the risk score positively correlates with the TMB level (R = 0.2, p = 5.9e-06). The median TMB level was utilized as the boundary to divide TMB into high and low groups. According to the K-M curve, the high TMB group has a better survival prognosis than the low TMB group (Figure 6E). When combined with the risk model, the low TMB+ high-risk group has the worst survival prognosis (Figure 6F).

### Tumor immune microenvironment estimate

**Figure 7:**
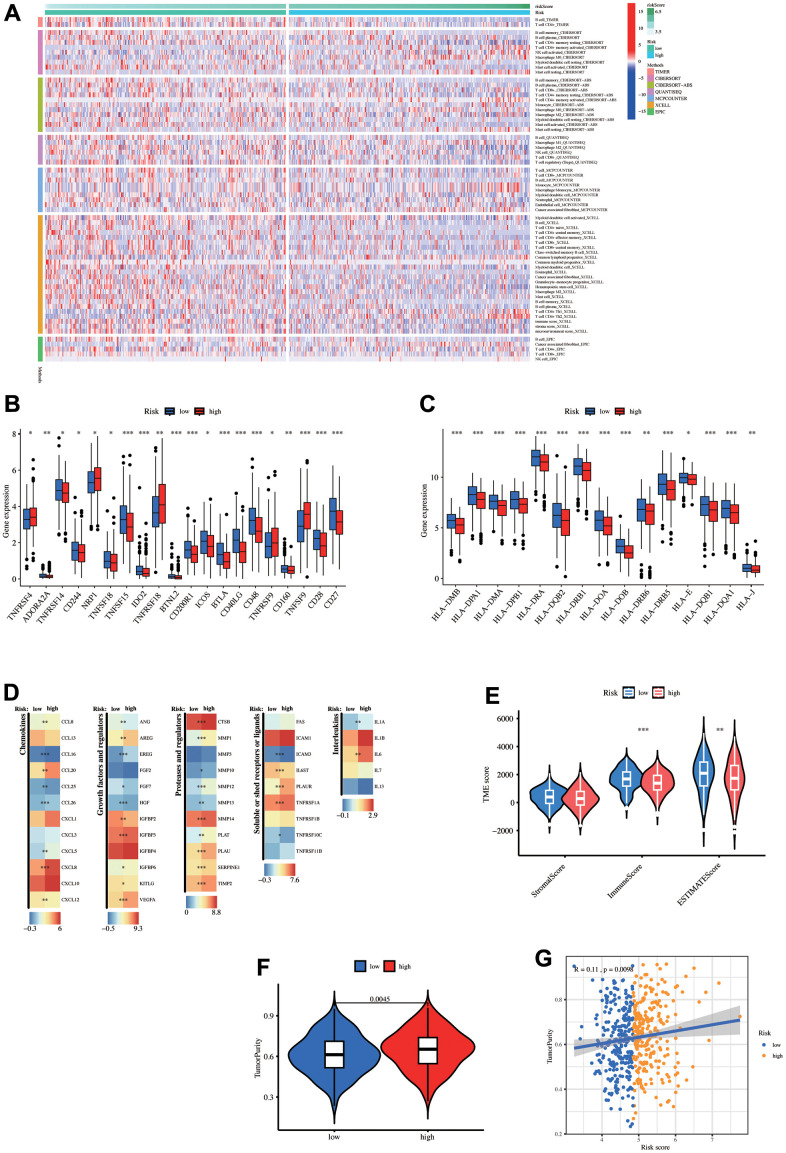
**Tumor microenvironment (TME) analysis.** (**A**) The heatmap displays the differences in the number of immune cells among the eight algorithms. Differential expression of (**B**) immune checkpoint-related genes, (**C**) MHC-related genes, and other (**D**) Tumor-related gene sets between high- and low-risk groups. (**E**) Estimate score differences between the high- and low-risk groups. (**F**) Tumor purity differences between the high- and low-risk groups. (**G**) Correlation between risk-score and tumor purity.

The immune cell composition and abundance in the TME strongly influenced tumor progression and immunotherapy effusiveness. A heat map demonstrates the immune cells with significant differences (p < 0.05, Figure 7A). Overall, the low-risk group had a significant abundance of immune cell infiltration, including T and B cells, in the TIMER and XCELL algorithms. Next, the common risk group immune checkpoint gene expressions were assessed (Supplementary Table 2). Twenty genes displayed significant differences, with 15 upregulated in the low-risk group and 5 upregulated in the high-risk group (Figure 7B). The differential expression of common MHC molecules was also analyzed (Supplementary Table 2). Interestingly, all 15 results with significant differences were high in the low-risk group (Figure 7B).

Moreover, the other tumor-related gene differential expression was analyzed (Figure 7C). The low-risk group had higher immune and estimate scores through the ‘estimate’ algorithm, but the stromal score did not differ significantly (Figure 7D). Supplementary Table 3 provides ‘estimate’ score details for each LUAD patient in the TCGA data. Moreover, tumor purity was reduced in the low-risk group (Figure 7F). The risk score and tumor purity exhibited a significant positive association (Figure 7G). In summary, the low-risk group had greater immune infiltration and upregulation of immune checkpoint-related and MHC genes.

### Immunotherapy efficacy prediction

**Figure 8:**
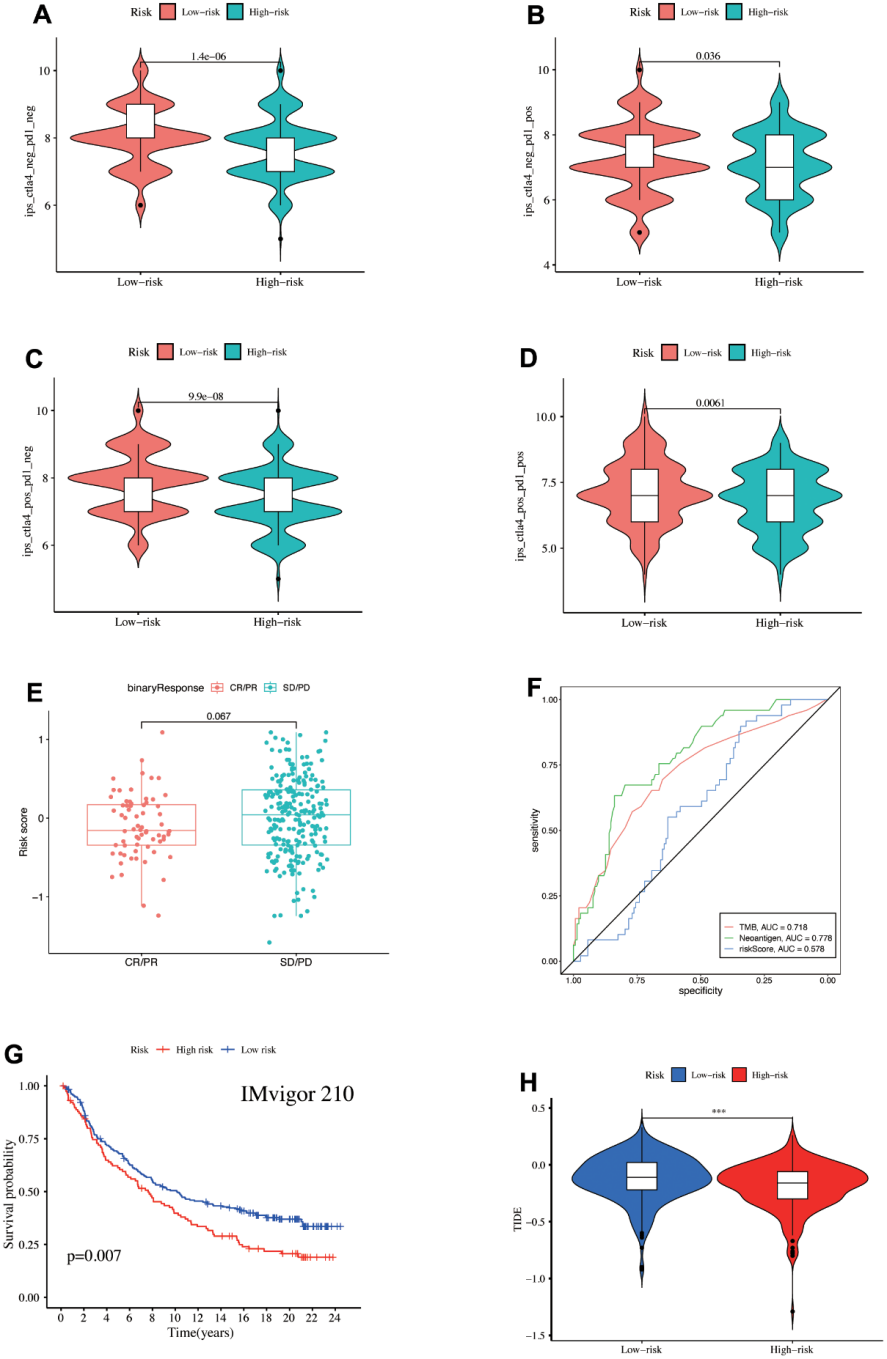
(**A**–**D**) IPS score differences. Validation of the model's ability to predict immunotherapy response in the IMvigor210 cohort, (**E**) Comparison of the risk score differences between CR + PR and SD + PD groups, (**F**) ROC curve illustrated the ability of TMB, Neoantigen, and risk-score to predict the efficacy of immunotherapy, (**G**) survival prognosis of high- and low-risk groups. (**H**) TIDE score differences in the TCGA database.

LUAD patient IPS was downloaded from the TCIA database to assess the immunotherapy response, indicating the higher IPS of the low-risk patients regardless of whether CTLA-4 and PD1 were expressed or not and further benefit from immunotherapy (Figure 8A–8D). Moreover, the complete response (CR) + partial response (PR) group risk score was decreased than the stable disease (SD) + progression disease (PD) group (Figure 8E), revealing the strongest diagnostic power of neoantigen (AUC = 0.778), followed by TMB (AUC = 0.718). The risk score also showed a certain predictive power (AUC = 0.578, Figure 8F). K-M analysis demonstrated that the high-risk patients had poor survival outcomes in the IMvigor210 immunotherapy cohort (Figure 8G). The low-risk patients had higher TIDE scores, indicating a high susceptibility to immune escape (Figure 8H). Our study discovered immunotherapy benefits for low-risk patients besides better long-term survival after immunotherapy.

### Prediction of chemotherapy response

**Figure 9:**
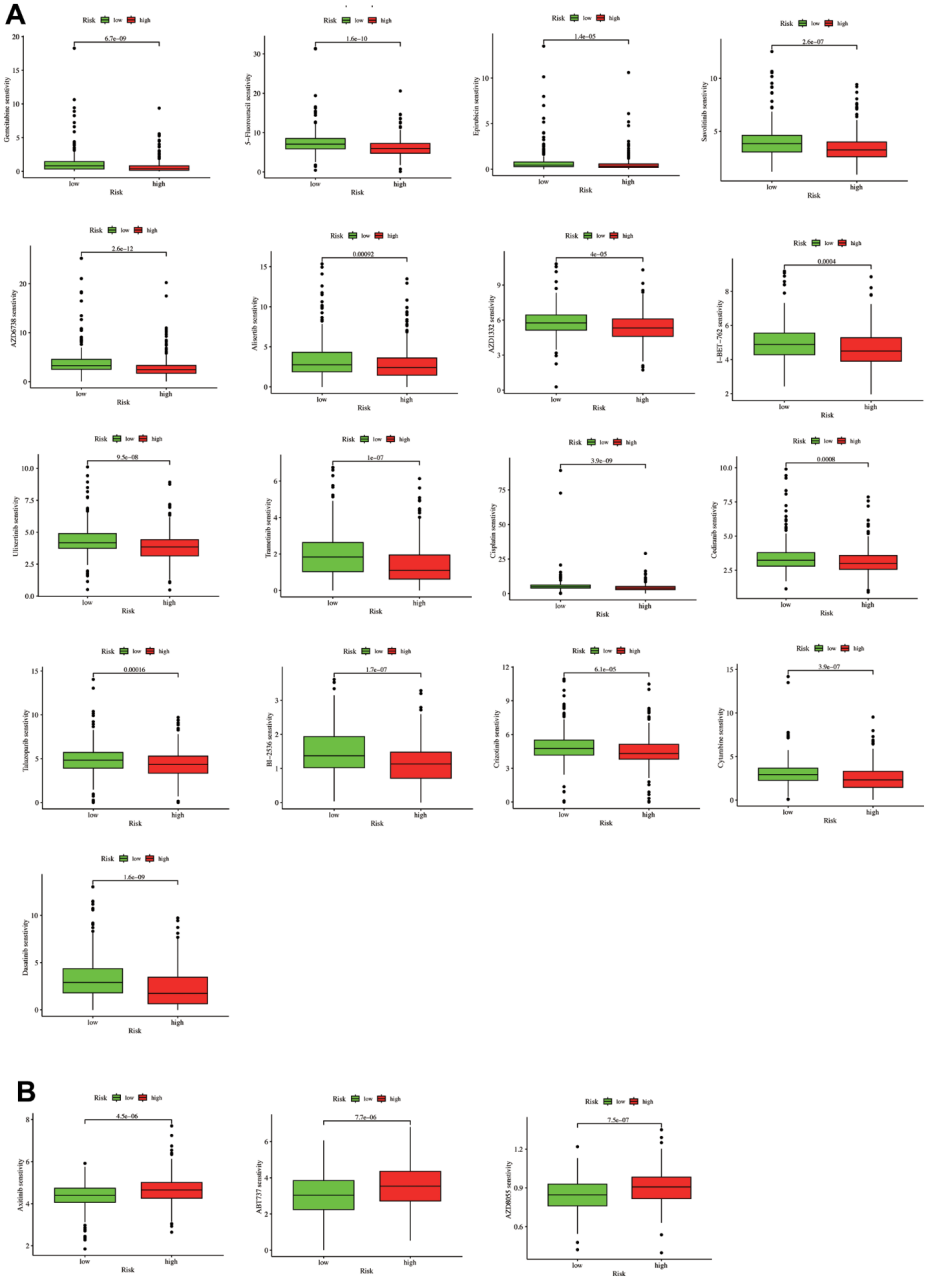
**A drug sensitivity analysis.** (**A**) Drugs with higher sensitivity in the low-risk group. (**B**) Drugs with higher sensitivity in the high-risk group.

Further analysis determined whether IC_50_ levels differed between the LUAD patient risk groups. We selected 20 LUAD-related chemotherapy drugs with significant differences in drug sensitivity, revealing that the low-risk group had high sensitivity to the following 17 drugs: Gemcitabine, 5-Fluorouracil, Epirubicin, Savolitinib, AZD6738, Alisertib, AZD1332, I-BET-762, Ulixertinib, Trametinib, Cisplatin, Cediranib, Talazoparib, BI−2536, Crizotinib, Cytarabine, and Dasatinib (Figure 9A). In contrast, three drugs (Axitinib, ABT737, and AZD8055) demonstrated higher sensitivity in high-risk patients (Figure 9B). The above results indicate that our model shows promising potential in distinguishing the sensitivity of LUAD patients to chemotherapy drugs, thereby providing new avenues for future treatment strategies in LUAD. However, further validation through clinical trials and animal experiments is necessary to confirm the accuracy of our drug sensitivity predictions.

## DISCUSSION

Lung cancer has the highest prevalence and mortality among all malignancies [1, 2]. LUAD, a primary subtype of lung cancer, has a five-year OS of less than 20% [38]. Immunotherapy has improved LUAD patient prognosis to a certain extent, but with some challenges, such as poor response and resistance [8, 9, 39]. Thus, a model that can predict LUAD prognosis and immunotherapy efficacy must be established. CAFs are the most important component of TME [11–15]. They can promote immunosuppressed TME by secreting cytokines, growth factors, chemokines, exosomes, and other methods, allowing tumors to escape the immune system [17]. Nevertheless, how CAFs are related to LUAD has been unrevealed, especially since the CAFs-related prediction model has yet to be established.

Our study constructed an eight-gene prognostic model based on CAFs-associated genes in combination with scRNA-Seq and bulkRNA-seq, demonstrating good prognostic and diagnostic capabilities in the TCGA database and four independent GEO validation cohorts. The high-risk group patient LUAD had a poor prognosis associated with a later pathological stage. Concurrently, eight genes involved in the model construction were also associated with LUAD pathogenesis or prognosis. The glycoprotein tissue inhibitor of metalloproteinase -1 (*TIMP-1*) primarily affects fibroblasts and keratinocytes proliferation and participates in ECM turnover [40, 41]. The interaction between *TIMP1* and *CD63* can promote LUAD progression [42], and *TIMP1* overexpression was correlated to poor LUAD prognosis [42, 43]. *TPM2* is an actin filament-binding protein whose primary function is stabilizing and integrating actin filaments [44, 45]. *TPM2* overexpression in LUAD may inhibit tumor proliferation and invasiveness, resulting in a better prognosis for patients [46]. Nuclear receptor subfamily 2 group F member 2 (*NR2F2*) is a nuclear orphan receptor primarily involved in tumor progression, stem cell differentiation, energy metabolism, and other processes [47, 48]. *NR2F2*-related signaling pathway can promote platinum resistance in lung cancer with brain metastasis via high glutathione (GLH) consumption [49]. The ECM protein Microfibrillar-associated protein 4 (*MFAP4*) locates primarily in the vascular wall ECM. It is involved in tissue repair and angiogenesis [50, 51]. *miR147b* can regulate *MFAP4* to promote LUAD progression [52]. Superoxide dismutase 3 (*SOD3*), a secreted antioxidant enzyme [53], primarily functions to maintain REDOX homeostasis in tissues [54]. LUAD has lower *SOD3* expression than normal tissues, and patients with elevated *SOD3* expression have a poor prognosis [55]. Caveolin-1 (*CAV1*) is a membrane protein predominantly involved in ECM remodeling, cell migration, cancer signaling, and endocytosis [56]. *CAV1*-derived peptides can inhibit idiopathic pulmonary fibrosis (IPF) progression [57], while *CAV1* overexpression can promote lung cancer progression and increase radiotherapy resistance [56]. The Serpin peptidase inhibitor clade H member 1 (*SERPINH1*) gene is a member of the serine proteinase inhibitor serpin superfamily [58]. *SERPINH1* is highly expressed in IPF and lung cancer patients, suggesting that it may be involved in both disease pathogenesis [59]. Flavin Containing Dimethylaniline Monooxygenase 2 (*FMO2*) gene is primarily present in mammalian lung tissues; its abundance in LUAD tissue is lower than in normal tissue [60].

Next, enrichment analyses were conducted to explore the biological roles of CAFs involved in LUAD. scRNA-seq analysis between CAFs cell subsets and 50 Hallmarkers revealed a positive correlation between CAFs and EMT and Wnt/β-catenin signaling. EMT is a cellular program that can promote tumorigenesis, metastatic tumor ability, and tumor resistance to anti-tumor therapy by reshaping cell-cell and cell-ECM interactions [61]. Wnt/β-catenin signaling is essential for embryonic development and tissue homeostasis. Abnormal WNT/β-catenin pathway activation is closely related to tumor progression and poor prognosis [62, 63]. Wnt/β-catenin signaling activation can regulate disease progression and metastasis in LUAD [64]. The enrichment analysis of 120 CAFs-related genes revealed a significant enrichment of these genes in ECM organization, protein digestion and absorption, and other ECM-related pathways. The enrichment analysis revealed significant enrichment in DNA replication and cell cycle pathways in the high-risk group, which may result in abnormal cell cycle regulation and DNA replication, leading to a poor prognosis. Meanwhile, the low-risk group had significant enrichment in immune-related pathways, such as the B cell receptor (BCR) signaling pathway, allograft rejection, and abundant immune cell infiltration, indicating a better prognosis.

The EGFR mutation group has higher risk scores. EGFR mutations are a common type of NSCLC mutation that affect approximately 40% to 55% of Asian LUAD patients [1, 65]. Although LUAD patients with EGFR mutations exhibited sensitivity to tyrosine kinase inhibitor (TKI) treatment, their overall prognosis was poor due to the tendency to develop drug resistance later in treatment [66, 67]. Patients with PD-L1 overexpression and EGFR mutations have a decreased immunotherapy response rate, possibly due to lower tumor-infiltrating T-cell activity in patients with EGFR mutations [68]. This is consistent with our IMvigor210 cohort study, where the low-risk group had better PD-1 therapy response and prognosis.

Afterward, the mutational landscape of the risk groups revealed a higher TP53 gene mutation frequency in the high-risk group (54% vs 38%). Patients with low TP53 mutation burden exhibited immune cell infiltration and were more probably to profit from immune checkpoint inhibitors (ICIs) therapy [69, 70]. In addition to TBM’s significant positive relation to the risk score and mutation-associated neoantigens (MANA), it was overexpressed in the high-risk group and involved in the CD8+T process of recognizing tumor cells during immunotherapy [37, 71]. It is assumed that high TMB-level patients exhibit more sensitivity to immunotherapy. However, TMB also has certain shortcomings in predicting the efficacy of immunotherapy. A study finding did not predict PFS, complete pathologic remission (CRP), and main pathological response (MPR) in stage IIIA NSCLC patients treated with immunotherapy [72]. Multiple factors, such as tumor antigens, lymphocyte infiltration, and antigen-presenting cells (APCs), influence tumor immunotherapy response [71]. It is a complex and dynamic process that needs comprehensive evaluation.

The TME difference was systematically analyzed between the two groups to explore whether the risk score can anticipate the immunotherapy response. Six different algorithms were employed to assess the differences in immune cell infiltration between the two groups, revealing a greater immune cell abundance in the low-risk patients. According to the TME evaluated by the ‘ESTIMATE’ algorithm, the high immune and estimate scores indicated the high immune cell infiltration that makes the tumor less likely to undergo immune escape, explaining this group’s better prognosis consisting of our enrichment analysis, thereby revealing its significant enrichment in immune-related pathways. In contrast, the high-risk group had higher tumor purity. Moreover, patients with high immune cell infiltration are more probably to profit from immunotherapy [73].

Furthermore, the relationship between risk score and immune-related genes revealed significant immune checkpoint-related gene abundance in the low-risk group. Meanwhile, all MHC molecules were significantly overexpressed in the low-risk group. T cells must recognize tumor antigens in MHC presence to generate anti-tumor immune responses. The immune checkpoint-related genes and the MHC gene overexpression were correlated to better immunotherapy response [74]. The TME analysis suggests the increased immunotherapy benefit for the low-risk group.

Subsequently, we utilized IPS [23] and TIDE [75] scores to predict immunotherapy response in the risk groups. We observed the performance of risk scores in immunotherapy cohorts (IMvigor210) treated with anti-PD-L1. Moreover, the IPS score correlates positively with immunotherapy efficacy [23]. We discovered that immunotherapy benefits patients in the low-risk group with higher IPS scores regardless of CTLA4 and PD1 expression. The CR + PR group patients had lower risk scores after IMvigor210 immunotherapy, indicating high immunotherapy sensitivity. Our risk score also had some predictive power for immunotherapy response (AUC = 0.578). In the IMvigor210 cohort, the high-risk group prognosis was poor, demonstrating the model accuracy and consistency for the LUAD patient prognosis prediction, consistent with the TCGA and GEO database results.

In contrast, the low-risk patients had a lower TIDE score and were more likely to have an immune escape. Despite having a lower TMB level and a higher TIDE score, the immune infiltration degree was higher, more immune-related pathways were enriched, and more immune-related genes were highly expressed. Finally, they had a better prognosis and immunotherapy effects. Therefore, higher immune cell infiltration level patients in LUAD are more likely to benefit from immunotherapy. A previous study revealed that the immune cell infiltration level was more predictive than the TMB level [21].

We also examined the drug susceptibility differences between the risk groups to better guide the risk score clinical practice. We revealed that the low-risk group had higher sensitivity to 17 drugs: Gemcitabine, 5-Fluorouracil, Epirubicin, Savolitinib, AZD6738, Alisertib, AZD1332, I-BET-762, Ulixertinib, Trametinib, Cisplatin, Cediranib, Talazoparib, BI-2536, Crizotinib, Cytarabine, and Dasatinib (Figure 9A). In contrast, three drugs (Axitinib, ABT737, and AZD8055) had higher sensitivity in high-risk patients.

We combined scRNA-seq and Bulk-RNA data to develop a predictive model of 8 CAFs-associated genes. Our model predicted LUAD prognosis and immunotherapy efficacy. The low-risk group patients had better survival prognoses and immunotherapy sensitivity. We also conducted enrichment, drug susceptibility, and mutational landscape analyses. We still have some drawbacks. Our analysis is a public database-dependent, and we need to conduct more *in vivo* and *in vitro* experiments. Some issues must be discussed in greater depth. In the future, we must continue to conduct in-depth study series to investigate the role of CAFs in LUAD more systematically and comprehensively and to provide innovative insights to treat LUAD precisely.

## Supplementary Material

Supplementary Figure 1

Supplementary Table 1

Supplementary Table 2

Supplementary Table 3
