# Supplementary material for: Prognostic analysis of lung adenocarcinoma based on cancer-associated fibroblasts genes using scRNA-sequencing
Source: Aging (Albany NY). 2023 Jul 11;15(14):6774–97. doi: 10.18632/aging.204838 (PMC10415565; doi:10.18632/aging.204838)
Supplement: Supplementary Figure 1 [file aging-15-204838-s001.pdf]

SUPPLEMENTARY FIGURE

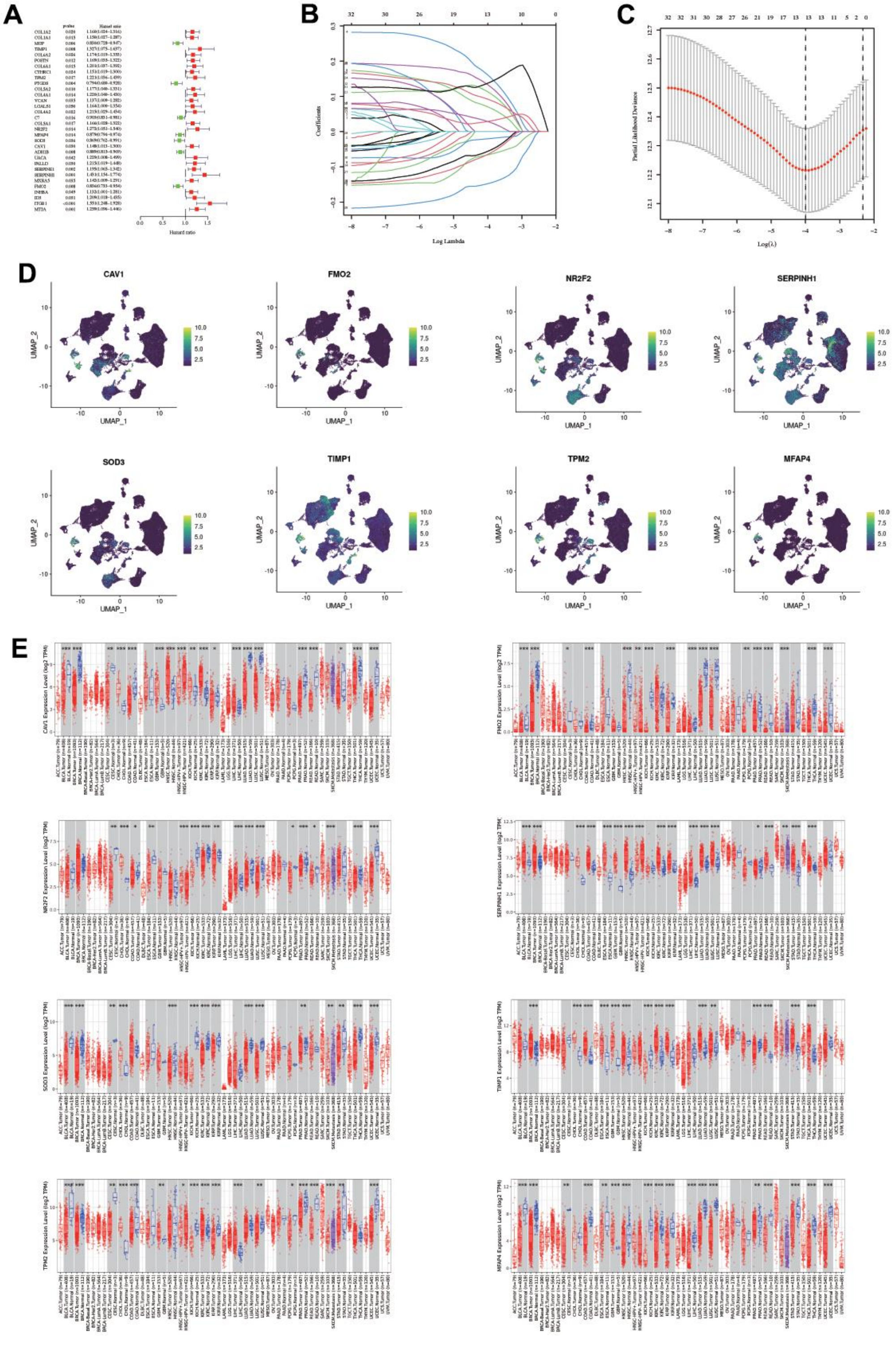

Supplementary Figure 1. (**A**) Genes with a significant impression on OS in univariate regression analysis. (**B**, **C**) Lasso regression analysis. (**D**) Distribution of eight CAFs-related genes involved in model construction in scRNA-seq. (**E**) Eight gene expressions were involved in model construction in the pan-cancer dataset.
