# Supplementary material for: Prognostic analysis of lung adenocarcinoma based on cancer-associated fibroblasts genes using scRNA-sequencing
Source: Aging (Albany NY). 2023 Jul 11;15(14):6774–97. doi: 10.18632/aging.204838 (PMC10415565; doi:10.18632/aging.204838)
Supplement: Supplementary Table 2 [file aging-15-204838-s003.pdf]

Supplementary Table 2. Information on 51 immune checkpoint-related genes and 23 MHC-related genes.

Checkpoints	MHC
PD-L1	HLA-E
PD1	HLA-DPB2
TIM3	HLA-C
CTLA4	HLA-J
B7-H4	HLA-DQB1
B7-H3	HLA-DQB2
BTLA	HLA-DQA1
VISTA	HLA-A
IDO1	HLA-DMA
PSGL-1	HLA-DOB
LAG3	HLA-DRB1
PD-L2	HLA-H
OX40	HLA-B
IDO2	HLA-DRB5
TNFRSF8	HLA-DOA
CD27	HLA-DPB1
ICOS	HLA-DRA
TNFRSF18	HLA-DRB6
TIGIT	HLA-L
TNFRSF9	HLA-F
TNFRSF14	HLA-G
TNFRSF4	HLA-DMB
CD28	HLA-DPA1
LGALS9	
CD70	
CD80	
LGALS9	
CD70	
CD80	
TNFSF15	
NRP1	
BTNL2	
HHLA2	
ICOSLG	
CD40	
TNFSF9	
TNFSF14	
CD86	
KIR3DL1	
CD200	
ADORA2A	
TNFRSF25	
CD244	
CD48	
LAIR1	

CD40LG
TMIGD2
CD200R1
TNFSF18
CD44
CD160
